# Ectopic Expression of T‐Box Transcription Factors Alters Mouse Forelimb Formation

**DOI:** 10.1002/dvg.70050

**Published:** 2026-04-14

**Authors:** Mariah H. Denhart, Deborah L. Chapman

**Affiliations:** ^1^ Department of Biological Sciences University of Pittsburgh Pittsburgh Pennsylvania USA

**Keywords:** Brachyury, limb, mouse, T, T‐box, Tbx6

## Abstract

The developing vertebrate forelimb expresses seven T‐box transcription factors, with several in overlapping expression domains. All T‐box transcription family members share similarity within their DNA binding domain, the T‐domain. Outside of the T‐domain, these factors share little similarity, allowing family members to have different transcriptional properties and binding partners. Several human T‐box genes show haploinsufficiency in the limb, including *Tbx5* and *Tbx3* that, when mutated, cause Holt‐Oram and ulnar‐mammary syndrome, respectively. This dosage sensitivity combined with the shared T‐domain leads to our hypothesis that when co‐expressed a competition between T‐box factors at target genes can occur. To test this, we ectopically expressed two exogenous T‐box factors, T and Tbx6, in the developing forelimb mesenchyme to examine how artificially changing the relative levels of T‐box proteins affects forelimb formation. Skeletal, apoptotic, and gene expression assays were used to characterize the resulting phenotypes. While ectopic T and Tbx6 both affected the size and shape of the forearm bones and ossification, they differentially affected digit formation: T caused loss of digits and Tbx6 led to phalange bone duplications and extra digit formation. These dissimilar phenotypes suggest that these transcriptional activators differentially affect pathways critical for regulating forelimb development.

## 1 Introduction

The DNA binding domain, the T‐domain, is the common feature of the T‐box transcription factors. *T* or *Brachyury* was the first to be identified, and mice with heterozygous mutations in T display a short‐tail phenotype (Dobrovolskaia‐Zavadskaia 1927). Sixteen additional T‐box proteins have been identified in mammals and play critical roles in development and disease progression [reviewed in (Papaioannou 2014; Ghosh et al. 2017; Li et al. 2024)]. The conserved T‐domain allows all family members to bind similar target sequences; however, outside of this domain there is little amino acid conservation, allowing differential activity of T‐box family members, with some acting as transcriptional activators, others as repressors, and still others possessing both activities. In vitro, T can bind to a palindromic sequence containing two consensus half‐sites, 5′‐**AGGTGT**GAAA‐3′ (core binding site sequence in bold) (Kispert and Herrmann 1993). Other T‐box family members can also bind to this half‐site, but their preferences for sequences surrounding the core sequence, spacing, and number of half‐sites vary between proteins (reviewed in Gentsch et al. 2017). Competition for DNA binding sites in tissues that express both activating and repressive T‐box proteins can be important for regulating proper levels of target gene expression. Farin et al. (2007) provided evidence that competition between Tbx18, a transcriptional repressor, and Tbx5, a transcriptional activator, is a possible mode of transcriptional regulation at the *Nppa* enhancer in cardiomyocytes. In the heart, Tbx2 represses *Nppa* expression while Tbx5 serves to activate; the mutually exclusive expression domains of Tbx2 and Tbx5 allow proper *Nppa* expression in the prechamber myocardium (Christoffels et al. 2004).

Seven T‐box genes are associated with mouse forelimb development: *Tbx2*, *Tbx3*, *Tbx5*, *Tbx15*, *Tbx18*, *Eomes*, and *T* (Chapman et al. 1996; Gibson‐Brown et al. 1998; Russ et al. 2000; Kraus et al. 2001; Liu et al. 2003; Singh et al. 2005). The *Tbx2* subfamily plays a major role in limb development and arose through a tandem duplication of an ancestral gene to originally create a linked gene pair which then underwent a duplication and dispersal event to create the *Tbx2–Tbx4* and *Tbx3–Tbx5* genes at separate chromosomal positions (Agulnik et al. 1996). In the limb, *Tbx5* and *Tbx4* are expressed in the mesenchyme of the fore‐ and hindlimb buds, respectively (Chapman et al. 1996; Gibson‐Brown et al. 1996). Both Tbx4 and Tbx5 are required for limb outgrowth but not limb identity (Minguillon et al. 2005). *Tbx2* and *Tbx3* are expressed in the anterior and posterior margins of both the fore‐ and hindlimbs with additional *Tbx3* expression in the apical ectodermal ridge (AER) (Chapman et al. 1996; Gibson‐Brown et al. 1996). Both Tbx2 and Tbx3 are transcriptional repressors (Carreira et al. 1998; Habets et al. 2002; Lingbeek et al. 2002). In humans, mutations in *TBX3* result in ulnar‐mammary syndrome and is characterized by malformation of the ulna and Digits 4 and 5 (Bamshad et al. 1997). Investigations into the forelimb phenotypes of conditional *Tbx2*/*Tbx3* double mouse mutants revealed more severe deformities than additive *Tbx2* and *Tbx3* single mutations; these forelimbs exhibited triplication of Digit 1, partial duplication of Digit 3, and loss of the ulna, Digit 4, and Digit 5, suggesting some partial redundancy and dosage sensitivity to Tbx2 and Tbx3 levels (Lopatka and Moon 2022). *Tbx5* expression can be detected in the lateral plate mesoderm as early as e8.5 (Agarwal et al. 2003; Minguillon et al. 2005). *Tbx5* acts as a transcriptional activator and is essential for initiating the Fgf‐loop that triggers forelimb outgrowth (Ahn et al. 2002; Ng et al. 2002; Rallis et al. 2003; Takeuchi et al. 2003). Tbx5 first activates *Fgf10* expression in the mesenchyme of the limb, which then activates *Fgf8* in the AER that signals back to the mesenchyme to maintain *Fgf10* expression (Ng et al. 2002; Hasson et al. 2007). If *Tbx5* expression is lost before the Fgf‐loop is initiated, the forelimb is not formed in the mouse, chick, or zebrafish (Ahn et al. 2002; Rallis et al. 2003; Takeuchi et al. 2003). Mutations in human *TBX5* affect limb outgrowth and are responsible for Holt‐Oram syndrome (Basson et al. 1997; Li et al. 1997). *Tbx15* and *Tbx18* are expressed in the core of the limb buds in overlapping domains (Agulnik et al. 1998; Kraus et al. 2001). *Tbx15* null mutants display defects in skeletal development; specific forelimb defects include shortening of humerus, radius, and ulna and the absence of central portion of the scapula, while limbs form normally in *Tbx18* mouse mutants (Singh et al. 2005; Farin et al. 2008). *Eomes* is expressed at the base of the fourth digit, although its function in limb development has not yet been elucidated (Hancock et al. 1999; Russ et al. 2000).

Our previous studies revealed that mouse T and Tbx6 are not functionally interchangeable (Wehn et al. 2020) and when ectopically expressed in the paraxial mesoderm each generates unique somite phenotypes (Wehn and Chapman 2010; Campbell et al. 2022). Ectopic Tbx6 expression results in somite phenotypes reminiscent of loss‐of‐function *Tbx15* and *Tbx18*, suggesting that ectopic Tbx6 competes with these endogenously expressed T‐box factors. Embryos ectopically expressing T possess small somites that appear to result from the migration of T‐expressing cells away from the paraxial mesoderm position. Interestingly, we further showed that this ectopic T expression led to *Tbx6* misexpression, which could also contribute to the observed phenotype (Campbell et al. 2022). Herein we tested the hypothesis that ectopic T and Tbx6 expression will interfere with endogenous T‐box factors but will do so differently and therefore will generate different limb phenotypes. Developmental syndromes in humans are associated with haploinsufficiency of several T‐box genes, suggesting that the level of T‐box proteins is important for normal development. As the developing vertebrate limb expresses several T‐box genes, some of which are expressed in overlapping regions, competition may be important for regulating normal limb development. Using the developing mouse limb as a model, we focused our analysis on the forelimb by examining how ectopic T or Tbx6 expression affected the forelimb skeleton; this included, from proximal to distal: the scapula, which is derived from the lateral plate and paraxial mesoderm (Huang et al. 2000; Valasek et al. 2011), humerus, radius and ulna, carpals, and phalanges. We also used marker gene analysis to determine whether critical limb signaling centers were impacted. Overall, we observed distinct forelimb malformation upon T or Tbx6 ectopic expression, but neither caused disruption to the critical signaling centers—the zone of polarizing activity (ZPA) or the AER.

## 2 Results

### 2.1 Transgenic Lines Used to Ectopically Express T and Tbx6

Two different transgenic systems were used to ectopically express T in the mouse limb. Our previously developed three‐component system, which utilized a Cre recombinase transgene that when expressed removed a floxed‐STOP cassette upstream of the reverse tet‐transactivator (rtTA) from a second transgene, allowing rtTA to act on the third transgene—a Tet‐responsive element controlling the expression of a myc‐tagged T (Campbell et al. 2022). This three‐component system enables finer temporal control with the added DOX requirement for rtTA activity. The two‐component system utilized a transgenic mouse line in which a floxed‐STOP cassette was positioned upstream of the mouse β‐actin promoter‐*T‐IRES‐GFP* inserted at the *ROSA26* locus. Cre recombinase removes the floxed‐STOP upstream of *T* in these embryos producing a chimeric T‐GFP protein. For all experiments, the *Prrx1‐Cre* transgenic line was used; the *Prrx1* enhancer drives Cre expression in the mesenchyme tissue of the forelimb beginning at e9.5 (Logan et al. 2002). Embryos must harbor both the *Prrx1‐Cre* and *floxed‐STOP‐βActin T* transgenes to express T‐GFP and are referred to as *Prrx1*‐*βAcT*; embryos without both transgenes were considered control littermates. Our previously described three‐component *Tbx6* transgenic line is similar to the *TRE‐myc‐T* transgene, requiring Cre recombinase, rtTA, and DOX for expression of myc‐tagged Tbx6 (Wehn and Chapman 2010). The *T* and *Tbx6* 3‐component embryos are referred to as *Prrx1*‐*T* or *Prrx1*‐*Tbx6*, respectively. Embryos not harboring all three transgenes are considered controls. Because expression of the *Prrx1‐Cre* is delayed in the hindlimb, our analysis focused on the forelimb bud phenotypes arising from the ectopic expression of T and Tbx6. For the three‐component systems, DOX administration began at e8.5, 1 day prior to the expected *Prrx1* enhancer activity.

### 2.2 Ectopic T or Tbx6 Expression Variably Affects Forelimb Development

**FIGURE 1:**
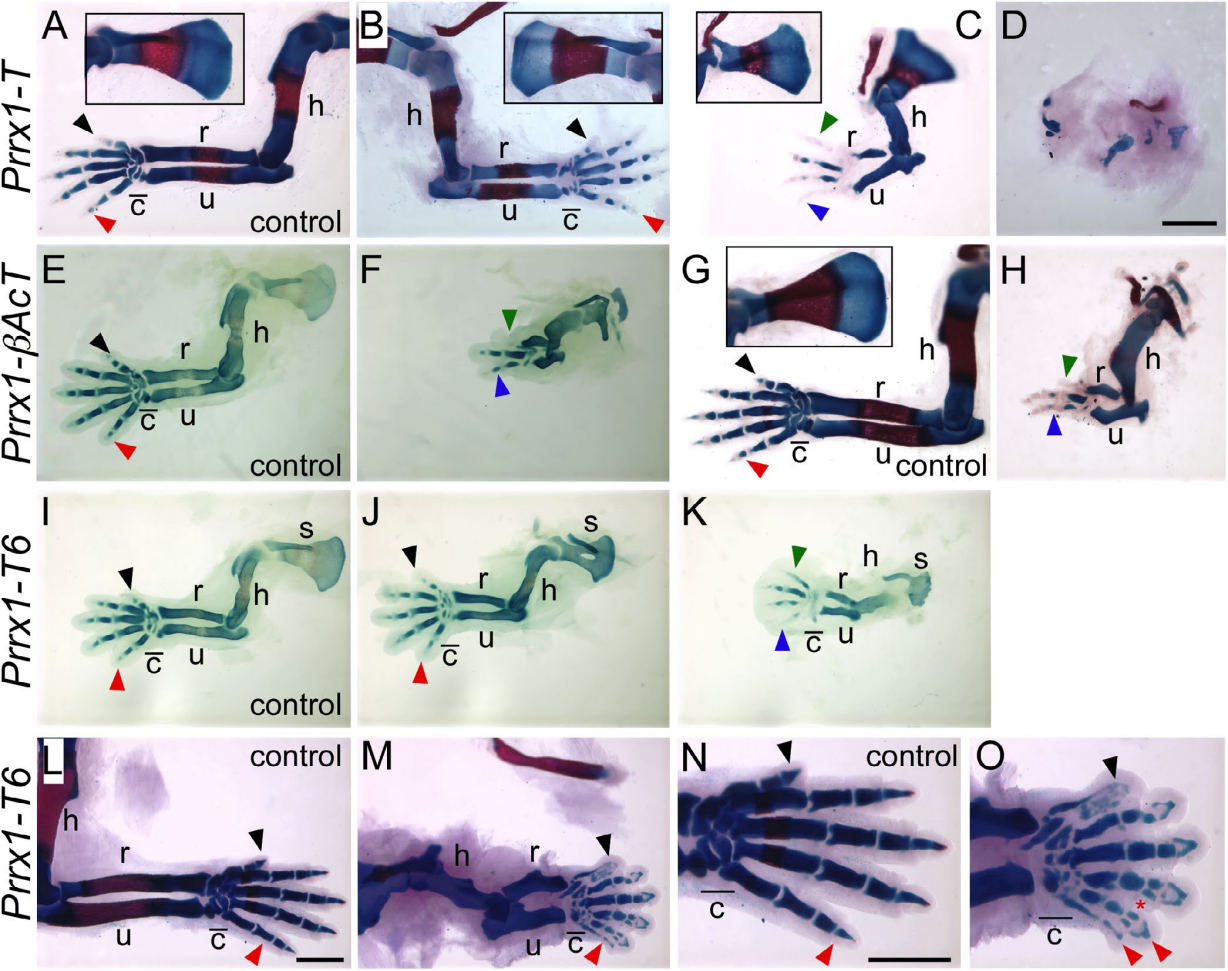
Ectopic T and Tbx6 expression result in variable limb skeletal deformities. Alcian blue and alizarin red staining of cartilage and ossified bone, respectively, of forelimbs at e16.5 (A–D, G, H) and e17.5 (L–O) and alcian blue staining of cartilage at e14.5 (E, F, I–K). All of the limbs are left limbs except for Panels (B, L–O), which are the right. Scapula insets are shown for Panels (A–C and G). Control (A) and *Prrx1‐T* (B–D) forelimbs fell roughly into the different phenotypes displayed (unaffected, similar to control in panel A to unrecognizable limbs). Reduced ossification (red stain) is observed in forelimbs for all three transgenics (C, H, M). Panels (N and O) are high magnifications of the distal limb elements shown in Panels (L and M), respectively. Control panels for each of the transgenics are indicated. c, carpals (c with line above; showing area of carpal formation); h, humerus; r, radius; s, scapula; u, ulna. Digit 1 is indicated by a black arrowhead, Digit 5 by a red arrowhead, and when digit identity cannot be ascertained, the most anterior digit is indicated by a blue arrowhead and the most posterior digit by a green arrowhead. The second red arrowhead in (O) indicates the duplication of Digit 5 and the red asterisk indicates a partial duplication of the intermediate phalange bone of Digits 4 and 5. Size bar in Panel (D) represents 1 mm and is the same for Panels (A–K); size bar in Panel (L) represents 1 mm and is the same for Panel (M); size bar in Panel (N) represents 1 mm and is the same for Panel (O).

**FIGURE 2:**
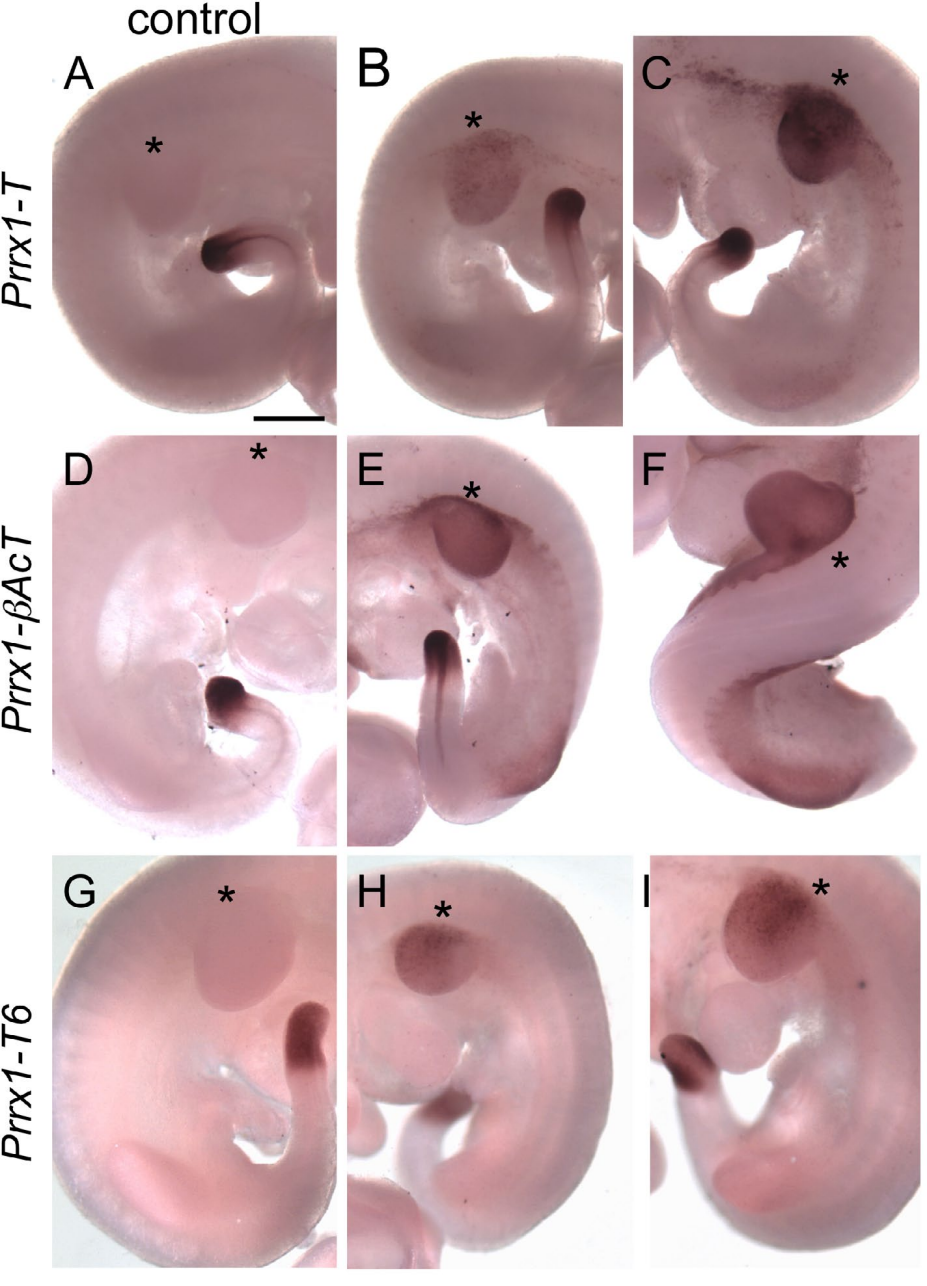
Ectopic expression of *T* and *Tbx6* in *Prrx1‐T*, *Prrx1‐βAcT*, and *Prrx1‐Tbx6* embryos. WISH of *T* (A–F) and *Tbx6* (G–I) expression in *Prrx1‐T* (B and C) and *Prrx1‐βAcT* (E and F) and *Prrx1‐Tbx6* (H and I) and their respective control embryos (A, D, G) at e10.5. Each genotype represents the variability of expression found within a single litter. An asterisk (*) in each panel is placed lateral to the forelimb bud. Endogenous *T* and *Tbx6* expression is detected in the primitive streak located in the tail of each embryo, with posterior notochord expression of *T* occasionally observed. Size bar in Panel (A) represents 500 μm and is the same for all panels.

Skeletal analysis of *Prrx1*‐*T* embryos at e16.5 revealed variable phenotypes, ranging from unaffected limbs to the loss of most of the forelimb elements (Figure 1A–D). Some general trends in *Prrx1*‐*T* skeletons include an overall smaller limb, including the scapula, highly reduced humerus, radius and ulna, missing radius, loss of carpals, and loss of several digits. Variability in skeleton deformities was not only seen between litters, but also within litters. This variability in skeletal deformities is likely due to the differences in the level of ectopic *T* expression between *Prrx1‐T* littermates (Figure 2A–C). No significant differences in phenotypes were observed between the left and right forelimbs (not shown).

*Prrx1*‐*βAcT* embryos showed less phenotypic variability than was observed with the three‐component system (Figure 1F,H); this is consistent with less variability in *T* expression (Figure 2D–F). At e14.5 and e16.5 *Prrx1*‐*βAcT* embryos had shortened forelimb elements that curved with only two to three digits formed at both stages [Figure 1 compare E and G (controls) to F and H (*Prrx1*‐*βAcT*)]. The two to three individual digits observed had reduced cartilage formation at both e14.5 and e16.5 (Figure 1F,H), and reduced ossification (red stain) of the digits as well as the humerus, radius, and ulna at e16.5 (Figure 1H).

Similar to *Prrx1*‐*T*, the *Prrx*‐*Tbx6* forelimbs also displayed variable phenotypes (Figure 1I–O), but not as extreme as those seen in *Prrx1*‐*T*. Variability in *Tbx6* expression in the three‐component embryos (Figure 2G–I) is in line with this range of phenotypes. At e14.5, *Prrx1*‐*Tbx6*, the humerus, radius, and ulna were shorter than controls, leading to the overall smaller forelimb, and had noticeably misshapen scapulae (Figure 1J,K). One embryo also displayed a hole in the scapula (Figure 1J). Nine of the 10 embryos analyzed lacked any cartilage condensation of Digit 1, and Digits 2–5 appeared underdeveloped (Figure 1K). At e14.5, carpal cartilage condensation appeared delayed, as most embryos had only one to two unique carpals compared to control embryos, which already had at least eight distinct carpals or lacked any distinct carpals [Figure 1, compare I (control) J and K (*Prrx1‐Tbx6*)]. At e16.5, *Prrx1*‐*Tbx6* embryos exhibited shortened bones and underdeveloped carpals (data not shown), but due to the fragility of the tissue and incomplete staining, digit defects were difficult to evaluate, and instead e17.5 forelimbs were assessed (Figure 1L–O). Embryos at e17.5 continued to show underdeveloped carpals, in addition to partial duplication of the intermediate phalange bone of Digit 5 in all four embryos assessed, with two of these embryos exhibiting a partial duplication of the intermediate phalange bone of Digit 4 as well (Figure 1M, and higher magnification Panel O). Digit 1, which was absent in most e14.5 embryos (Figure 1K), was present in e17.5 limbs, but was improperly developed, as the phalange bones appeared as many small fragments instead of identifiable bones (Figure 1M,O). A duplication of a digit, presumably Digit 5, was also observed (Figure 1M,O). The distal phalange bones of Digits 2–5 appeared “V”‐shaped instead of a solid triangular shape, and while the distal tips are separated, the rest of the digits are still connected by interdigit mesenchyme (Figure 1M,O). Finally, the ossified areas in the central regions of the phalanges have expanded outwards in control embryos but are absent in the *Prrx1*‐*Tbx6* embryos [Figure 1, compare N (control) and O (*Prrx1‐Tbx6*)].

### 2.3 Changes in Apoptosis Pattern in *Prrx1‐βAcT* and *Prrx1‐Tbx6* Embryonic Forelimbs

We next explored whether increased apoptosis could account for the changes in limb morphology. A possible cause for the loss of skeletal element in *Prrx1*‐*T* limbs could be increased cell death before digits condense. Since changes in *Prrx1*‐*T* limb morphology were not apparent until e12.5 and later, LysoTracker Red staining was performed at e12.5. Lysotracker staining did not reveal any remarkable differences in cell death in *Prrx1*‐*T* forelimbs at e12.5 (data not shown).

**FIGURE 3:**
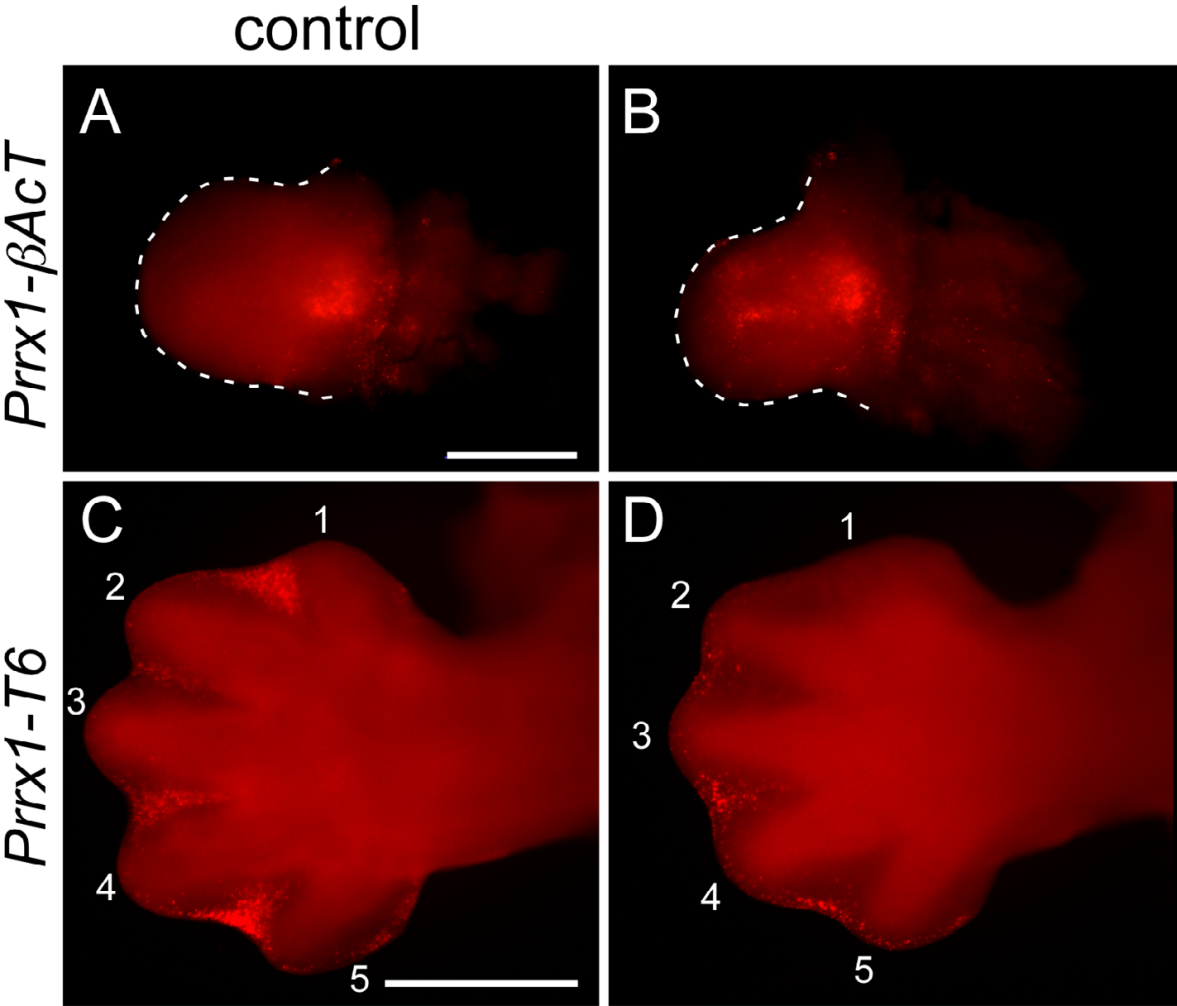
Apoptosis changes in *Prrx1‐βAct* and *Prrx1‐Tbx6* embryos. Lysotracker Red staining of the dorsal side of the right forelimb buds from *Prrx1‐βAcT* (A and B) and *Prrx1‐Tbx6* (C and D) embryos at e10.5 and e13.5, respectively. The forelimbs are outlined in Panels (A and B). The digits are numbered 1–5 in Panels (C and D). Anterior is the top and posterior the bottom in each panel. (A and B) Apoptotic cells are observed in the proximal position of control (A) and *Prrx1‐βAcT* (B) forelimbs, with additional apoptotic cells in the more distal anterior area of *Prrx1‐βAcT*. Less Lysotracker Red staining is observed between Digits 1 and 2 and Digits 4 and 5 in the *Prrx1‐Tbx6* forelimb (D) compared to the control (C). Size bar in panel A represents 500 μm and is the same for Panel (B). Size bar in Panel (C) represents 500 μm and is the same for Panel (D).

**FIGURE 4:**
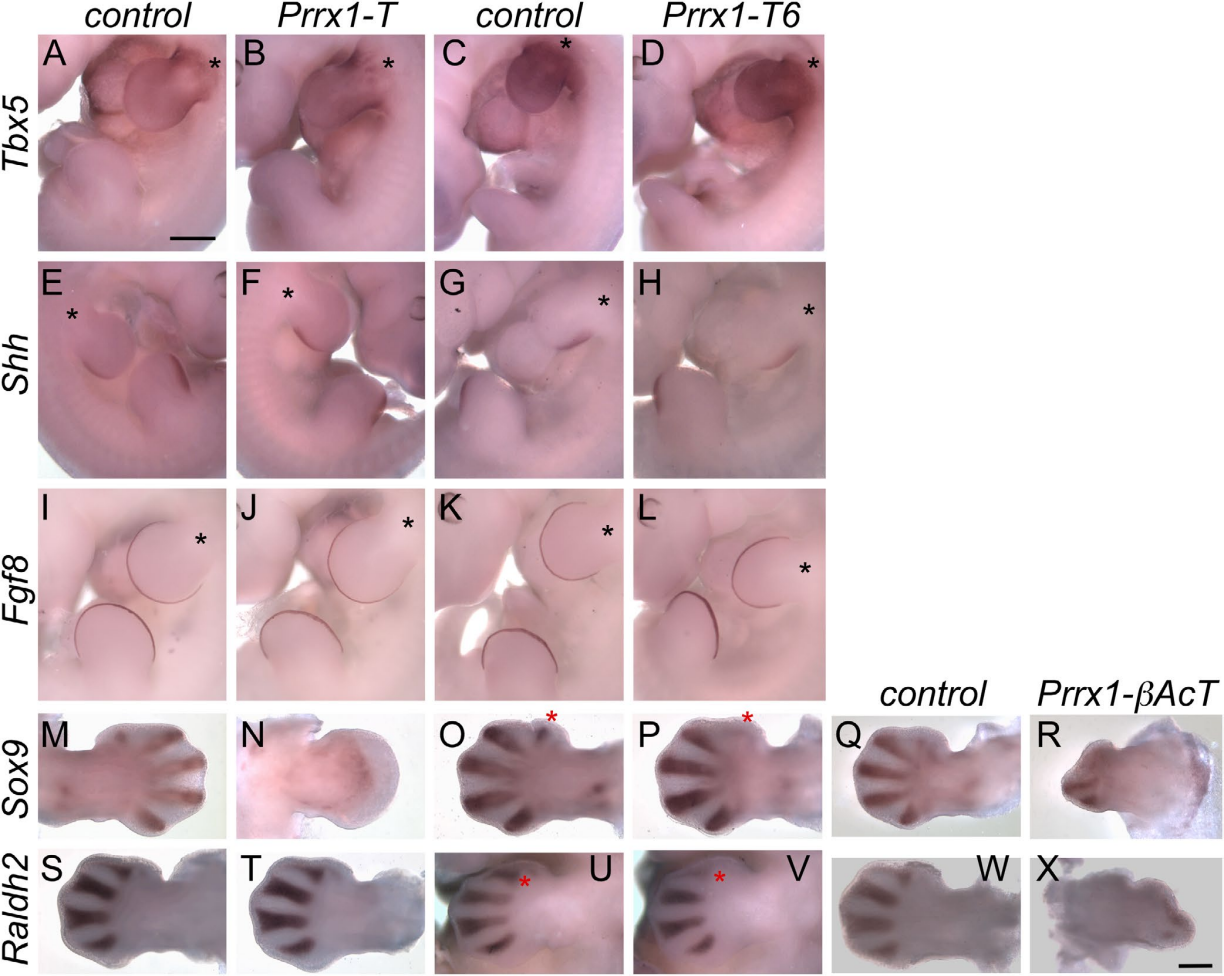
WISH analysis of forelimb marker gene expression. WISH for each marker gene listed: (A–D) *Tbx5*, (E–H) *Shh*, and (I–L) *Fgf8* expression at e11.5 in *Prrx1‐T* and *Prrx1‐Tbx6* embryos to the right of their respective control littermates. (M–R) *Sox9* and S‐X *Raldh2* expression at e12.5 in *Prrx1‐T*, *Prrx1‐Tbx6*, and *Prrx1‐βAcT* forelimbs to the right of their respective control littermates. An asterisk (*) in Panels (A–L) is placed medial to the forelimb bud. A red asterisk (*) in Panels O and P indicates the Digit 1 position. A red asterisk (*) in Panels (U and V) indicates interdigit regions 1–2. The dorsal side of the right (E, F, M, N, X) or the left (A–D, G–L, O–W) limb bud is shown. Size bar in panel A represents 500 μm and is the same for Panels (A‐L). Size bar in Panel (B) represents 500 μm and is the same for Panels (M–X).

Interdigit tissue in the mouse autopod undergoes apoptosis starting at e13.0 and is finished by e14.0 to produce individual digits (Wanek et al. 1989; Martin 1990; Salas‐Vidal et al. 2001). Only two to three digits are apparent at e14.5 in *Prrx1‐βAcT* embryos (Figure 1F). Unlike *Prrx1*‐*T* embryos which often maintain a paddle‐shaped limb bud in early development, *Prrx1*‐*βAcT* embryos often exhibit a slightly less rounded paddle shape at e11.5 that becomes increasingly angular in the anterior digit forming region by e12.5 (see forelimbs WISH analysis, Figure 4R,X). A possible cause of this change in shape is cell death occurring in the digit forming region. Lysotracker staining revealed increased apoptosis at the distal anterior region of the limb bud at e10.5 (Figure 3B).

Much of the interdigit region of severely affected *Prrx1*‐*Tbx6* embryos was intact at e14.5 compared to controls which already exhibited individualized digits (Figure 1K). Apoptosis was therefore analyzed by Lysotracker staining at e13.5. These forelimbs showed a decrease in apoptotic cells particularly in the interdigit region between Digits 1–2 and 4–5 (Figure 3, compare Panels C (control) and D (*Prrx1‐Tbx6*)).

### 2.4 Limb Signaling Centers Largely Unaffected in T and Tbx6 Expressing Embryos

To elucidate the underlying cause of the skeletal phenotypes observed upon ectopic *T* or *Tbx6* expression in the various transgenic lines, we performed marker gene expression analysis by whole‐mount in situ hybridization (WISH). The skeletal phenotypes observed were similar to those of *Tbx5* hypomorphic embryos, specifically shortening of the humerus and defects in digit formation (Sulaiman et al. 2016). We therefore examined *Tbx5* expression, but no difference in *Tbx5* forelimb expression was observed in control versus experimental e11.5 forelimbs for either *Prrx1‐T* or *Prrx1‐Tbx6* (Figure 4A–D). The developing limb contains signaling centers critical for outgrowth and patterning, specifically the AER and the ZPA (Towers and Tickle 2009). The AER, the thickened ectoderm at the distal tip of the limb buds, promotes outgrowth of the limb bud through an Fgf‐feedback loop (Crossley et al. 1996; Towers and Tickle 2009). This loop is initiated by fibroblast growth factor 10 (Fgf10) in the mesenchyme signaling to the AER, which activates *Fgf8* expression that then signals back to the mesenchyme to maintain *Fgf10* expression (Xu et al. 1998). Once this pathway is active, the ZPA is established in the posterior region of the limb bud; the ZPA is critical for anterior–posterior patterning of the limb (Riddle et al. 1993). Previous research suggested that *T* may have a role in positively regulating *Fgf10* expression and maintaining the AER (Liu et al. 2003). We hypothesized that forelimb expression of T in the *Prrx1*‐*T* and *Prrx1‐βAcT* embryos could result in changes to the AER or ZPA. Neither *Shh*, a marker of the ZPA, nor *Fgf8*, a target of *Fgf10* and a marker of the AER, showed differences in expression at e11.5 in control versus experimental *Prrx1*‐*T* forelimbs (Figure 4E,F,I,J). Similarly, forelimb expression of *Tbx5*, *Shh*, and *Fgf8* was indistinguishable for both *Prrx1‐βAcT* (data not shown) and *Prrx1‐Tbx6* forelimbs compared to controls (Figure 4C,D,G,H,K,L).

Decreased expression of *Sox9*, a marker of chondrogenesis (de Crombrugghe et al. 2000), was observed in the digit forming region of the *Prrx1*‐*T* and *Prrx1*‐*βAcT* forelimb buds compared to controls at e12.5 (Figure 4M,N,Q,R). The interdigit marker *Raldh2* (Zhao et al. 2010) did not show a change in expression in the *Prrx1*‐*T* embryos (Figure 4S,T) but was detected in the malformed *Prrx1*‐*βAcT* forelimbs (Figure 4W,X).

In e12.5 *Prrx1*‐*Tbx6* forelimbs, *Sox9* expression was absent in the Digit 1 position (Figure 4, compare O and P), while *Raldh2* expression was decreased in the interdigit region between Digits 1 and 2 (Figure 4, compare U and V). *Raldh2* expression in the other interdigit regions appears unaffected (Figure 4U,V). *Tbx15* and *Tbx18* are thought to have redundant functions in ossification (Farin et al. 2007). Because delayed ossification was observed in *Prrx1‐Tbx6* forelimb digits by e14.5, *Tbx18* expression was evaluated; however, at e11.5 there were no obvious differences in *Tbx18* expression observed in control versus experimental forelimbs (data not shown).

## 3 Discussion

Ectopic expression of *T* and *Tbx6* in the limb bud resulted in distinct limb phenotypes in the transgenic embryos (summarized in Table 1). Forelimb phenotypes for both *Prrx1‐T* and *Prrx1‐Tbx6* were highly variable, which was likely due to the variable levels of *T* and *Tbx6* expression in the early limb bud (Figure 2). The wide variability in phenotypes even within litters has been previously observed in three‐component T and Tbx6 embryos (Wehn and Chapman 2010; Campbell et al. 2022) and is likely associated with the levels of ectopic *T* and *Tbx6* expressed. The more uniform ectopic *T* expression in the *Prrx1‐βAcT* can be attributed to requiring only the removal of the STOP of transcription/translation upstream of the *β‐Actin* promoter to drive *T* expression, while the three‐component system also requires DOX. The three‐component system will only express T/Tbx6 when the floxed‐STOP is removed upstream of the *rtTA* and DOX is available to enable rtTA to activate the tet‐responsive element to then drive *T/Tbx6* expression. DOX was delivered orally to the pregnant females via oral gavage 1 day prior to the *Prrx1* enhancer being active and thereafter via water bottle administration. While the amount of DOX consumed can vary depending on the female's water intake, the littermate embryos should presumably be exposed to the same level of DOX. None‐the‐less, differences in *T* and *Tbx6* expression are observed in three‐component embryo littermates. *Prrx1‐βAcT* exhibited more uniform limb phenotypes, which overlapped with those observed for *Prrx1‐T*. Interestingly, despite *Prrx1‐βAcT* delivering more consistent *T* expression (Figure 2E,F), the skeletal defects were never as severe as the most severe deformities in *Prrx1‐T* (Figure 1D compare to F).

**TABLE 1:** Summary of forelimb phenotypes arising from ectopic expression of T and Tbx6.

Transgene	Limb phenotype first observed	Early patterning genes (e11.5): Tbx5, Fgf8, Shh	Later patterning genes (e12.5): Sox9, Raldh2	Apoptosis (time examined)	Skeletal phenotypes (time examined)
*Prrx1‐T*	e12.5	No change	*Sox9* decreased digit forming region; raldh2 no change	No change (e12.5)	Shortened forelimb elements (scapula, humerus, radius, ulna); loss of radius, carpals, digits (e16.5)
*Prrx1‐βAcT*	e11.5	No change	*Sox9* and *Raldh2* decreased digit forming region	Increased apoptosis distal anterior (e10.5)	Shortened forelimb elements (scapula, humerus, radius, ulna); loss of 2‐3 digits; reduced cartilage and reduced ossification of forelimb elements (e14.5, e16.5)
*Prrx1‐Tbx6*	e13.5	No change	*Sox9* absent digit 1; *Raldh2* decreased interdigit 1‐2	Decreased apoptosis interdigit 1 and 4 (e13.5)	Shortened forelimb elements (humerus, radius, ulna, scapula); reduced or missing digit 1 and carpals; partial duplication of digits 4 and 5; “v‐shaped” phalange at distal tips; reduced ossification (e14.5, e17.5)

Limb morphological differences for the majority of *Prrx1‐T* forelimbs were not obvious until e12.5 or later. While we did not observe differences in cell death at e12.5, the loss of digits and limb elements (Figure 1B–D) could be caused by increased cell death at later stages. Because *Prrx1‐T* forelimbs do not have obvious phenotypes until e12.5, it is not surprising that expression of *Tbx5*, *Shh*, and *Fgf8* is similar to control forelimbs. *Prrx1‐βAcT* embryos exhibit smaller forelimb buds by e11.5. Lysotracker revealed increased cell death in the limb bud by e10.5. This increased cell death may account for the reduced‐sized limb bones, absence of carpal elements, and absence of digits at e14.5 and e16.5 in *Prrx1‐βAcT* forelimbs.

*Sox9* expression is decreased in *Prrx1‐T* and *Prrx1‐βAcT* forelimbs suggesting that chondrogenesis is impaired. *Raldh2* expression was reduced in *Prrx1‐βAcT* e12.5 forelimbs; however, these limbs already had obvious misshaped phenotypes. *Prrx1‐T* forelimb buds utilized for assaying *Raldh2* expression showed no apparent phenotypic differences or changes to *Raldh2* expression. The absence of an effect on *Raldh2* expression could be because these embryos simply expressed low levels of ectopic T expression.

Ectopic Tbx6 expression in *Prrx1‐Tbx6* forelimbs resulted in shorter limbs, abnormal Digit 1 formation, partial duplications of phalanges of Digits 4 and 5, and duplication of Digit 5. *Prrx1‐Tbx6* forelimbs showed ossification defects of the limb bones and Digit 1 (Figure 1I–O). Like *Prrx1‐T* forelimbs, *Prrx1‐Tbx6* forelimbs showed no difference in *Tbx5*, *Shh*, or *Fgf8* expression. *Prrx1‐Tbx6* forelimbs do not appear different from control littermates until e12.5‐e13.5. At e14.5, Digit 1 cartilage is missing, and the forelimb bud remains paddle‐shaped. Lysotracker at e13.5 revealed a decrease in interdigit apoptosis between Digits 1 and 2 and Digits 4 and 5 (Figure 3C,D), with interdigit tissue remaining at e17.5 where only the distal digit tips were separated while the remaining more proximal digit regions still appeared to be connected by mesenchymal tissue (Figure 1O).

In e12.5 *Prrx1*‐*Tbx6* forelimbs, *Sox9* expression was noticeably absent in the Digit 1 position (Figure 4P), which could explain the absence of Digit 1 at e14.5 (Figure 1K). By e17.5 cartilage did form in the Digit 1 region, but these appeared fragmented rather than distinct phalange bones. Furthermore, *Raldh2* expression was decreased in the interdigit region between Digits 1 and 2 (Figure 4, compare U and V). As retinoic acid is implicated in apoptosis of the interdigit region (Zhao et al. 2010), loss of expression of *Raldh2*, the major enzyme producing retinoic acid, in this region could explain the decreased apoptosis in *Prrx1‐Tbx6* in at least interdigit 1–2 region but does not explain the decreased apoptosis in interdigit 4–5 region (Figure 3D). *Tbx15* and *Tbx18* are thought to have redundant functions in ossification (Farin et al. 2007). Because delayed ossification was observed in *Prrx1‐Tbx6* forelimbs, *Tbx18* expression was evaluated, but at e11.5 there was no difference in *Tbx18* expression observed in control versus experimental limbs (data not shown). Ectopic expression of Tbx6 in the forelimb resulted in a hole in the scapula of one of nine embryos examined (Figure 1J). Interestingly, ectopic expression of Tbx6 in the paraxial mesoderm consistently led to a hole in the scapula, which we previously connected to possible competition of Tbx6 with Tbx15 as the *Tbx15* null shares this trait. The similarities of *Prrx1 Cre‐TRE‐Tbx6* (forelimb) and *Dll Cre‐TRE‐Tbx6* (paraxial mesoderm) are likely due to dual origin of the scapula from both paraxial and lateral plate mesoderm.

The partial duplication of phalangeal bones of *Prrx1‐Tbx6* Digits 4 and 5 is reminiscent of forelimbs lacking *Tbx2* and *Tbx3*. A previous study linked the loss of *Tbx2* and the subsequent delay in termination of the Shh and Fgf pathways with a larger limb bud and the resulting partial Digit 4 duplication (Farin et al. 2013). In *Prrx1‐Tbx6* embryos the forelimb buds do not appear to be larger than control littermates, but the lack of apoptosis between the digits may present a similar environment for partial duplications to occur.

We hypothesized that ectopic T or Tbx6 expression could compete with endogenous T‐box factors for binding at target genes. If T or Tbx6 were able to effectively compete with Tbx5, we expected to see phenotypes like those observed for *Tbx5* null embryos in which forelimbs are not formed (Rallis et al. 2003). Our phenotypes only overlap with those observed for *Tbx5* hypomorphic limbs created using a gene deletion‐gene replacement strategy (Sulaiman et al. 2016), suggesting that T or Tbx6 can inefficiently compete with endogenous Tbx5. The consensus binding site sequences for T and Tbx5 are 5′‐AGGTGTGAAA‐3′ and 5′‐(A/G)GGTGT(C/G/T)(A/G)‐3′, respectively, as determined by in vitro selection assays, indicating that while Tbx5 could bind to the T consensus site, T did not bind to the Tbx5 consensus site (Ghosh et al. 2001). Marker gene analysis by WISH did not reveal differences in the expression of *Tbx5*, *Shh*, and *Fgf8* in the forelimb bud for *Prrx1‐T*, *Prrx1‐βAcT*, or *Prrx1‐Tbx6* further suggesting that neither ectopic T nor Tbx6 expression can compete at the expression levels achieved in our transgenic systems. *Fgf10* is a direct downstream target of Tbx5 and the site that elicits the highest activity by Tbx5 is GTGGGA (Agarwal et al. 2003), which could explain the absence of Tbx5 interference by T which would manifest as a change in the expression of Fgf‐loop genes. Another possible explanation is that the level of T expressed is not high enough to disrupt Tbx5's initiation of the Fgf‐loop.

Ectopically expressing *T* or *Tbx6* resulted in abnormal forelimb phenotypes that share similarities with loss of function mutations of various T‐box genes. However, the *Prrx1‐T* and *Prrx1‐Tbx6* phenotypes are not equivalent, suggesting that if some level of competition between exogenous and endogenous T‐box factors is occurring, then T and Tbx6 differentially compete with these endogenous factors at target genes. Differential competition can be due to binding affinities or activities of T and Tbx6 at target genes. For example, Tbx6 is a much stronger activator of the *Dll1* enhancer than T in vitro (Wehn et al. 2020). Alternatively, the different phenotypes could be due to a different selection of target genes in this ectopic area expression area based on their different binding affinities and/or the presence of co‐factors. In cardiomyocytes, Tbx5 and Tbx18 can both interact with Nkx2.5 and GATA4, which raises the possibility that competition between these Tbx5 and Tbx18 for binding to these proteins could contribute to transcriptional regulation of *Nppa* (Farin et al. 2007). In the absence of knowing the identity of all T‐box binding partners, competition for co‐factors cannot be completely ruled out. The most likely mode of action, however, is competition at the level of DNA binding since the T‐domain, the DNA binding domain, is the only region of conservation between family members. During mouse gastrulation, Eomesodermin can block T function in cells where they are co‐expressed based on their binding specificity (Schule et al. 2023). The *Prrx1‐T* and *Prrx1‐Tbx6* phenotypes are difficult to interpret because of the number of T‐box factors expressed in the area of T/Tbx6 ectopic expression. Complicating the interpretation of these different phenotypes are the variable levels of T/Tbx6 ectopic expression and the range of phenotypes observed. A simpler system that more consistently drives ectopic expression of T/Tbx6 in a tissue endogenously expressing only one T‐box factor would help to clarify the mechanism underlying the phenotypes observed.

## 4 Materials and Methods

### 4.1 Mice

*TRE‐myc‐T* and *TRE‐myc‐Tbx6* mice have previously been described (Wehn and Chapman 2010; Campbell et al. 2022). The *Prrx1‐Cre* (Logan et al. 2002) (*B6.Cg‐Tg* ^*(Prrx1‐cre)1Cjt/J*^, Jax stock #005584), *βActin T IRES‐GFP* (*Gt(ROSA)26* ^*Sortm1(Actb‐T,‐GFP)Dalco*^, Jax stock #024179) and *ROSA26‐reverse tet‐transactivator* (*R26‐rtTA*) (*B6.Cg‐Gt(ROSA)26* ^*Sortm1*^ *(rtTA, EGFP)* ^*Nagy*^ */J*, Jax stock #005670) transgenic lines were obtained from Jackson Laboratory. Mice were mated and checked daily for the presence of a copulation plug. Noon on the day of the plug was considered embryonic day (e) 0.5. Doxycycline hydrochloride (DOX) was administered via oral gavage at 2.5 mg/kg and was added to the drinking water [2 mg/mL DOX, plus sucrose (50 mg/mL)] for pregnant females beginning at e8.5 and continuing until embryos were harvested at designated time points. Control embryos for the *βAcT* 2‐component embryos did not carry the Cre transgene, while controls for the *Prrx1‐T* and *Prrx1‐Tbx6* 3‐component embryos did not carry all three transgenes (*TRE*, *rtTA*, *Cre*). All animal work was performed in accordance with the guidelines established by the University of Pittsburgh's Institutional Animal Care and Use Committee.

### 4.2 Whole‐Mount In Situ Hybridization

Whole‐mount in situ hybridization (WISH) was performed as previously described (Wilkinson et al. 1990) using antisense riboprobes for *T* (Wilkinson et al. 1990), *Tbx6* (Chapman et al. 2003), *Tbx5* (Chapman et al. 1996), *Fgf8 (*Crossley and Martin 1995), *Shh* (Chang et al. 1994), *Sox9 (*Bi et al. 1999), *Raldh2* (*Horizon MMM1013‐202858355*), and *Tbx18* (Kraus et al. 2001). Hybridizations and washes were performed at 63°C. Number of experimental embryos examined for each probe: *T* (*Prrx1‐T*: 7; *Prrx1‐βAcT*: 3), Tbx6 (*Prrx1‐T6*: 7), Tbx5 (*Prrx1‐T*: 3; *Prrx1‐T6*: 2); *Fgf8* (*Prrx1‐T*: 3; *Prrx1‐βAcT*: 2; *Prrx1‐T6*: *3*); *Shh* (*Prrx1‐T*: 7; *Prrx1‐βAcT*: 2; *Prrx1‐T6*: *3*); *Sox9* (*Prrx1‐T*: 6; *Prrx1‐βAcT*: 3; *Prrx1‐T6*: *2*); *Raldh2* (*Prrx1‐T*: 3; *Prrx1‐βAcT*: 2; *Prrx1‐T6*: *1*); *Tbx18* (*Prrx1‐T6*: 3).

### 4.3 Skeleton Preparations

Skeletons from e13.5‐e17.5 embryos were stained with alcian blue with or without alizarin red as previously described (Nagy et al. 2003), except that the staining was performed at 37°C. Alcian blue stained embryos were cleared in benzyl benzoate: benzyl alcohol while alcian blue/alizarin red skeletons were cleared in glycerol.

### 4.4 Cell Death Assay

Cell death was assayed using LysoTracker Red staining essentially as previously described (Fogel et al. 2012). Briefly, embryos were dissected in Hanks' Balanced Salt Solution (HBSS), incubated with LysoTracker Red DND‐99 (Invitrogen) for 45 min at 37°C, washed in HBSS, and fixed in 4% paraformaldehyde overnight at 4°C. Embryos were washed in HBSS, progressively dehydrated in a methanol series, and stored in 100% methanol at −20°C until they were imaged. Embryos were rehydrated through a methanol:PBT series, incubated with DAPI (1:3000) for 1 h at room temperature in PBT, and then washed in PBT (1× PBS, 0.1% Triton X‐100). Nof experimental embryos analyzed (*Prrx1‐T*: 6; *Prrx1‐βAcT*: 9; *Prrx1‐T6*: 9).

## Funding

This work was supported by the National Science Foundation (Grant IOS 1050189 2011).

## Data Availability

The transgenic animals utilized have previously been described in other publications or are available commercially.
